# Mobile health applications: awareness, attitudes, and practices among medical students in Malaysia

**DOI:** 10.1186/s12909-022-03603-4

**Published:** 2022-07-15

**Authors:** Julian Valerie John Jembai, Yi Lin Charlene Wong, Nur Alia Muhammad Amir Bakhtiar, Siti Nursuraya Md Lazim, Hwei Sung Ling, Pei Xuan Kuan, Pin Fen Chua

**Affiliations:** 1 Faculty of Medicine and Health Sciences, Universiti Malaysia Sarawak, Kota Saramahan, Sarawak, Malaysia; 2 Digital Health Research and Innovation, Institute for Clinical Research, National Institutes of Health Malaysia, Shah Alam, Malaysia

**Keywords:** Applications, COVID-19, Health and fitness, Medical education, Mobile health

## Abstract

**Background:**

The popularity of mobile health (mHealth) applications (or apps) in the field of health and medical education is rapidly increasing, especially since the COVID-19 pandemic. We aimed to assess awareness, attitudes, practices, and factors associated with the mHealth app usage among medical students.

**Methods:**

We conducted a cross-sectional study involving medical students at a government university in Sarawak, Malaysia, from February to April 2021. Validated questionnaires were administered to all consenting students. These questionnaires included questions on basic demographic information as well as awareness, attitude toward, and practices with mHealth apps concerned with medical education, health and fitness, and COVID-19 management.

**Results:**

Respondents had favorable attitudes toward mHealth apps (medical education [61.8%], health and fitness [76.3%], and COVID-19 management [82.7%]). Respondents’ mean attitude scores were four out of five for all three app categories. However, respondents used COVID-19 management apps more frequently (73.5%) than those for medical education (35.7%) and fitness (39.0%). Usage of all three app categories was significantly associated with the respondent’s awareness and attitude. Respondents in the top 20% in term of household income and study duration were more likely to use medical education apps. The number of respondents who used COVID-19 apps was higher in the top 20% household income group than in the other income groups. The most common barrier to the use of apps was uncertainty regarding the most suitable apps to choose.

**Conclusion:**

Our study highlighted a discrepancy between awareness of mHealth apps and positive attitudes toward them and their use. Recognition of barriers to using mHealth apps by relevant authorities may be necessary to increase the usage of these apps.

**Supplementary Information:**

The online version contains supplementary material available at 10.1186/s12909-022-03603-4.

## Background

The World Health Organization’s Global Observatory defines mobile health (mHealth) as a public health practice supported by mobile devices such as mobile phones, patient monitoring devices, personal digital assistants, and other wireless devices [1]. Generally, mHealth is used to provide healthcare information to the public, collect health data, monitor patients remotely, access health records, make medical diagnoses, and assist in disease prevention and management [2, 3]. They are generally classified as medical education and teaching apps, health and fitness apps for patients and the public, telemedicine and telehealth apps, and others [4].

Medical education apps are emerging technologies for training and practice in healthcare services [5]. These educational applications for medical providers involve medical terminologies and functions, including drug-referencing and clinical decision-support tools [4]. Various clinical and teaching apps are designed for medical students and health professionals in the field of medical education. These apps enhance clinical knowledge, aid decision-making, provide telemedicine, and facilitate patient care [5–7]. Drug and clinical reference apps, diagnostic apps, and medical calculators are common mHealth apps used by healthcare professionals and students. Health professionals find these apps particularly useful for accessing the latest medical journals during patient care and management [8]. These apps also help in rapidly accessing critical information during clinical practice and bedside learning [9]. Given their valuable functions, mHealth medical education apps are likely to be widely adopted in every aspect of clinical practice [8].

Health and fitness mHealth apps promote healthy lifestyles, particularly in adolescents and students [10]. Common features of fitness apps include step counters, calorie counters, weight loss management functions, and exercise regimes. The use of these apps for home-based exercise is encouraged and expected to increase, especially in the ongoing COVID-19 pandemic, when movement outside the home is restricted [11, 12]. The integrated use of wearable devices and mHealth apps increases motivation and practice efficacy among users seeking healthier lifestyles [13].

The COVID-19 pandemic has led to the development of COVID-19 information and symptom-tracking apps. COVID-19 management apps provide users with information on the health measures to be taken based on their recorded symptoms [14]. Such apps have also revolutionized public health monitoring. One such COVID-19 monitoring app uses Bluetooth technology to send push notifications, to alert those in the vicinity individuals with possible COVID-19 infection [14]. Such apps provide the public with information regarding the disease, good hygiene practices, and standard operating procedures (SOPs) [15].

MHealth applications are used by all age groups such as maternal-child groups and older adults [16, 17]. During the COVID-19 pandemic, these applications improved the healthcare accessibility and streamlined in-hospital practice workflow and public health systems [18, 19]. In addition to healthcare providers, mHealth also empowered patients in term of disease management, especially in self-management and remote monitoring of chronic illness [20]. Populations in lower to middle-income areas benefit from mHealth interventions through effective healthcare deliveries, which is later reflected by better clinical outcomes [21]. Governments that developed mHealth in lower-income areas have indirectly promoted equity of healthcare access by addressing the gaps in limited resources, such as internet accessibility [22, 23].

MHealth technology has helped healthcare workers (HCWs) improve the efficiency of their work in public healthcare or in-hospital settings. Most HCWs, including community HCWs or even midwives, applied mHealth for data collection during patient visits and communicating with peers or patients, especially during community visits and population surveillance [24]. However, HCWs, as role models for embracing this technology have poor adoption and face the challenges in encouraging the public usage of mHealth [25]. Thus, training programs for improving clinicians’ mHealth skills have been proposed [26]. Medical schools are also expected to revise their curricula and incorporate mHealth within their syllabus. Early exposure of medical students to mHealth could ensure its adoption when they join the workforce. An immediate example of mHealth usage among medical students is the use of mobile learning to replace physical education during the COVID-19 pandemic. This usage is expected to expand to several aspects of medical school programs.

Despite the vast evidence in the literature highlighting the benefits of mHealth, poor public adoption remains a challenge. Various patterns of mHealth usage have been found among different demographics [27]. Socioeconomic factors were found to be one barrier to the adoption [28]. Medical students also face challenges in using these technologies [29]. Medical student who are the future medical workforce, are expected to excel in their professional training and embrace emerging competencies such as incorporating data and technology in healthcare service. Reviews show that medical schools are beginning to redesign the curriculum to meet the need of the 21st century, and the COVID-19 pandemic could be the catalyst that transforms and hastens this process [30]. The pandemic is expected to transform the teaching methods of medical schools by integrating telemedicine [31]. Hence, more studies on mHealth usage, its perception, and associated barriers among medical students are needed to provide vital references for authorities such as medical curricular teams. In this study, we aimed to investigate the awareness about, attitude toward, and practice of using mHealth apps among medical students. After a non-exhaustive literature review, to the best of our knowledge, ours is the first study on this topic in Malaysia. We hope that our results expand the scarce information in this regard in Southeast Asia.

In tandem with the fourth industrial revolution (4IR), mHealth apps have become essential in the rapidly evolving field of digital healthcare. Despite increasing usage of mobile phones in Malaysia, public knowledge regarding the use of mobile for health care is lacking. To date, little is known on the awareness of medical students’ awareness, attitudes, and practices and the obstacles to the adoption of mHealth during the COVID-19 pandemic. Thus, we aimed to assess awareness about, attitudes toward, and practices of mHealth apps usage among medical students at the Universiti Malaysia Sarawak (UNIMAS). The outcome of our study will provide mHealth app providers, medical educators, and health institutions with insights that will allow them to improve mHealth adoption.

## Methods

### Sample population and questionnaire design

This cross-sectional study administered a questionnaire to 739 undergraduate medical students (214 males and 525 females) from UNIMAS to assess their awareness, attitudes, and practices with mHealth apps. Of the 739 undergraduate medical students, 301 were in their preclinical years (year 1 and 2), whereas the remaining 438 were in their clinical years (year 3 to 5). The inclusion criterion for the respondents was medical students from UNIMAS with access to digital devices. Convenience sampling was used in this study. The sample size was determined using the open-source calculator Open-Source Epidemiology Statistics for Public Health (Open Epi) v. 3.01. To attain a confidence level of 95%, a sample size of 247 respondents was required. Allowing for a potential 10% attrition rate, a minimum number of 275 respondents were required. Ethical approval was obtained from the Ethics Committee of UNIMAS Faculty of Medicine and Health Sciences and the Malaysian National Medical Research Register (NMRR) (NMRR-20–2834-57,731), and the Medical Research and Ethics Committee [ref: KKM/NIHSEC/P21-67(4)].

The questionnaire was developed based on two primary relevant papers [28, 32] and other existing literature [33–37]. The questionnaire comprised four sections: Sect. 1 covered social demographics; Sect. 2 covered awareness of, attitudes toward, and practices with medical education mHealth apps; Sect. 3 covered awareness and attitudes; and Sect. 4 covered the use of mHealth fitness and health apps and awareness of, attitudes toward, and practices with COVID-19 mHealth apps. Sections 2 to 4 assessed students’ attitudes using a Likert-type scale (scores ranging from 1 to 5). Experienced content experts evaluated each item on the questionnaire for content validity. Face validity was determined using five medical students via video conference due to the social distancing mandate during the COVID-19 pandemic. The questionnaire was subsequently piloted among 12 medical students.

Questionnaire items on medical, health and fitness, and COVID-19 mHealth apps were assessed for internal consistency using Cronbach’s alpha (CA). The questionnaire showed good reliability with a CA of 0.651 for questions on medical apps, 0.777 for questions on health and fitness apps, and 0.907 for questions on COVID-19 apps.

### Data collection

A structured, standardized questionnaire including questions that assessed awareness of, attitudes toward, and practices with mHealth usage were administered to medical students between February 3, 2021, and April 25, 2021. All participants were provided with a subject information sheet and were briefed. After obtaining written consent, the students were given an internet link to access the self-administered questionnaire.

### Measures

#### Outcome variables

The respondents’ awareness and use of mHealth apps were measured in Sects. 2 to 4 in a binary fashion as “aware or not aware of mHealth apps” and “use or do not use mHealth apps.” Respondents’ attitudes toward the three categories of apps were scored in Sects. 2 to 4 using a Likert scale with scores ranging from 1 to 5. The mean (average) attitude score of Sects. 2 to 4 was calculated for each respondent.

#### Exposure variables

Basic demographic information for all participant was collected including age (years), gender (male or female), ethnicity (Malay, Chinese, Indian, Sarawak Bumiputera/indigenous group, or others), household income (bottom 40[B40]: < RM4849 per month, middle 40 [M40]: RM4850–10,959 per month, or top 20 [T20]: > RM10960 per month], phase of medical training (clinical or preclinical), mobile device ownership (yes or no), and type of device (smartphone, tablet, laptop, or wearable device). The classification of household income by the Department of Statistics Malaysia was included to assess the respondent’s family financial status [38]. We also evaluated the frequency of use for each app (at least once a day, at least once a week, at least once a month, or less than once a month). For medical and fitness apps, duration of use was also assessed (less than 6 months, 6 months to 2 years, or > 2 years).

### Statistical analyses

Statistical analyses were performed using SPSS Statistics for Windows v. 24.0 (IBM Corp., Armonk, N.Y., USA). All demographic information and outcome (in categorical data format) are presented as frequencies (n) and percentages (%), except the mean attitude score, is presented as a continuous variable. Categorical variables were subjected to univariate analysis using the chi-squared test (*χ*2), whereas continuous variables were analyzed with an independent t-test. Fisher's exact test was used when the expected count in a cell was < 5. *P-*values of < 0.05 were considered statistically significant.

## Results

### Demographic profiles of participants

A total of 249 valid questionnaires were returned. The demographic characteristics of the respondents are shown in Table 1. The average age of the respondents was 21 years, and the majority were female (68.7%). Respondents were undergraduate medical students distributed roughly evenly between the clinical (53%) and preclinical (47%) phases of study. The respondents were ethnically diverse. Most were in the M40 group (49.4%), with a family income between RM4850 and RM10959. Smartphones (99.2%) and laptops (95.6%) were the most owned gadgets. Two-thirds of the respondents had > 20 Gb as their monthly telephone company (Telco) data plan.

**Table 1:** Demographic characteristics of respondents

***Variables***	***Responses***	***Frequency, n***	***Percentage (%)***
**Age**	19	16	6.4
	20	53	21.3
	21	53	21.3
	22	55	22.1
	23	42	16.9
	24	21	8.4
	25	9	3.6
**Gender**	Male	78	31.3
	Female	171	68.7
**Ethnicity**	Malay	89	35.7
	Chinese	71	28.5
	Indian	18	7.2
	Sarawak Bumiputera	62	24.9
	Others	9	3.6
**Phase of Study**	Preclinical	117	47.0
	Clinical	132	53.0
**Own mobile device**	Yes	249	100.0
	No	0	0
**Type of Device**	Smartphone	247	99.2
	Tablet	138	55.4
	Laptop	238	95.6
	Wearable devices	31	12.4
**Household income**	< RM4849 per month (B40)	77	30.9
	< RM4850–10,959 per month (M40)	123	49.4
	RM10,960 per month (T20)	49	19.7

### Overall awareness of, attitudes toward, and use of mobile health applications among the respondents

As shown in Table 2, most respondents (82.7%) were aware of the existence and function of COVID-19 apps, followed by personal health and fitness apps (76.3%) and medical education apps (61.8%). The same trend was observed in positive attitudes (“agree” and “strongly agree”) toward the three types of applications: COVID-19 management apps (total mean score: 83), personal health and fitness apps (75.7), and medical education apps (70.6). However, the trend did not persist in respondents’ app usage. Despite the high adoption of COVID-19 applications (73.5%), the use of health and fitness (39%) and medical education apps (35.7%) were much lower. My Sejahtera (99.5%), a locally authorized COVID-19 management app; physical activity tracking apps (89.7%); and the medical education app Medscape (92.1%) were the most used applications.

**Table 2:** Awareness of, attitudes toward, and overall usage of mobile health applications among study respondents

**Variable**	**Aware**				**Frequency, n**					**Percentage (%)**		
**Respondents’ awareness of mHealth apps**												
**for medical education**	**Yes**				154					(61.8)		
	**No**				95					(38.2)		
**for health and fitness**	**Yes**				190					(76.3)		
	**No**				59					(23.7)		
**for COVID-19 management**	**Yes**				206					(82.7)		
	**No**				43					(17.3)		
**Respondents’ attitudes toward mHealth apps**												
**Statements**	Strongly disagree (%)		Disagree (%)		Neutral (%)		Agree (%)			Strongly agree (%)		Mean score
***Medical education apps***												
**Information on medical education apps is reliable**	1.2	1.2			28.5	0.0				69.1		4.3
**Medical education apps are easy to use**	0.8	1.6			23.7	51.4				22.5		4.0
**Medical education apps are not costly**	3.2	15.3			28.9	31.3				21.3		3.5
**Medical education apps are a useful source of medical information**	2.4	0.8			15.7	51.0				30.1		4.2
**Medical education apps save time searching for medical information**	2.8	2.4			18.5	49.8				26.5		3.9
**Total mean score**												**4.0**
***Health and fitness apps***												
**Health and fitness apps are easy to use**	1.2			1.2	15.3	59.4				22.9		4.0
**Health and fitness apps are not costly**	2.4			8.4	23.7	38.6				26.9		3.8
**Health and fitness apps are useful for health improvement**	0.8			1.2	26.1	42.6				29.3		4.0
**Health and fitness apps save my time records and track my health data**	0.8			1.2	14.9	49.0				34.1		4.1
**Total mean score**												**4.0**
***COVID-19 management apps***												
**COVID-19 app information is reliable**	0.8			1.2	14.9	57.4			25.7		4.1	
**Apps are easy to use**	0.8			1.2	12.4	56.2			29.3		4.1	
**Apps are not costly**	0.8			0.8	10.8	44.6			43.0		4.3	
**Apps are useful for pandemic control**	1.2			1.2	16.5	44.6			36.5		4.1	
**Apps save time (e.g., scan-in to enter premises, record local health risk, and check the latest COVID-19 information)**	0.8			0.8	9.2	46.6			42.6		4.5	
**User privacy is protected when using the apps**	1.6			1.2	25.7	42.6			28.9		4.0	
**Total mean score**											**4.2**	
**Respondents’ use of mHealth apps**												
**Variable**	**Responses**				**Frequency, n**			**Percentage (%)**				
**Apps installed and used to seek medical information**	Yes				89			(35.7)				
	No				160			(64.3)				
**Medical education app(s) used**	UpToDate				21			(23.6)				
	Medscape				82			(92.1)				
	DynaMed				8			(9.0)				
	Oxford Clinical Handbook				31			(34.8)				
	Epocrates				16			(18.0)				
	Others				44			(49.4)				
**Apps installed and used for health and fitness purposes**	Yes				97			(39.0)				
	No				152			(61.0)				
**Health and fitness app(s) used**	Physical activity				87			(89.7)				
	Food trackers				38			(39.2)				
	Fasting apps				18			(18.6)				
	Others				2			(2.1)				
**Apps installed and used for COVID-19 management**	Yes				183			(73.5)				
	No				66			(26.5)				
**COVID-19 management app(s) used**	My Sejahtera				182			(99.5)				
	COVID trace				16			(8.7)				
	Qmunity				33			(18.0)				
	My Trace				4			(2.2)				

### Obstacles to mHealth application use among respondents

As shown in Table 3, most respondents (up to 73.5%) were uncertain of the most suitable mHealth app to use. Around one-third of the respondents identified personal budget and data privacy concerns as hindrances to app usage. Notably uncertainty regarding app usage (37.5%) and lack of confidence in the information they provide (21.3%) were also the barriers to mHealth app usage in our study.

**Table 3:** Obstacles to use of mHealth applications

**Obstacles to mHealth app use**	**Frequency n (%)**
**Medical education apps**	
** Do not know how to assess medical information using mHealth apps**	60 (37.5)
** Do not know which app(s) is/are most suitable**	110 (68.8)
** Do not trust information provided by the app(s)**	34 (21.3)
** Limited budget for app subscription**	47 (29.4)
**Health and fitness apps**	
** Do not know which health app(s) is most suitable**	112 (73.7)
** Concerns regarding the privacy of personal health data**	40 (26.3)
** Limited budget for app subscription**	35 (23.0)
**COVID-19 management apps**	
** Do not know how to use the app(s)**	27 (40.9)
** Do not know which COVID-19 app(s) is/are most suitable**	37 (56.1)
** Do not want to disclose personal information**	11 (16.7**)**

### Relationships between respondent demographics and mHealth app awareness, attitudes, and practices

As expected, the respondents’ awareness of the three categories of mHealth apps significantly affected their usage (Table 4). Higher usage of mHealth app was observed when the respondents were aware of such apps. Of the respondents who did not use the apps, 59.1% were unaware of their existence or function.

**Table 4:** MHealth app awareness and use

**Respondents’ awareness of apps**	**Use of medical education apps**		***p-*** **value**	**Use of health and fitness apps**		***p-*** **value**	**Use of COVID-19 management apps**		***p-*** **value**
	**Yes**	**No**		**Yes**	**No**		**Yes**	**No**	
	***n*** ** = ** ***89 (%)***	***n*** ** = ** ***160 (%)***		***n*** ** = ** ***97 (%)***	***n*** ** = ** ***192 (%)***		***n*** ** = ** ***183 (%)***	***n*** ** = ** ***66 (%)***	
**Aware**	87 (97.8)	67 (41.9)	.00	95 (97.9)	95 (62.5)	.00	179 (97.8)	27 (40.9)	.00
**Not aware**	2 (2.2)	93 (58.1)		2 (2.1)	57 (37.5)		4 (1.2)	39 (59.1)	

The mean attitude scores were different for those who used the apps and those who did not (Table 5). This was true for all three categories of mHealth apps. A higher mean attitude score translated to higher adoption of apps. COVID-19 apps were used the most (74%) and had the highest mean attitude score (5.45).

**Table 5:** Associations between mean scores for attitudes toward mHealth apps and overall app usage

**Types of mHealth apps used.**	**mHealth app usage**	**n (%)**	**Mean attitude score**	***p-*** **value**
**Medical education apps**	Yes	89 (36)	4.25	.00
	No	160 (34)	3.64	
**Health and fitness apps**	Yes	97 (39)	4.18	.00
	No	152 (61)	3.18	
**COVID-19 management apps**	Yes	183 (74)	4.28	.00
	No	66 (26)	3.75	

We analyzed the relationships between respondent demographics, including gender, learning phase, household income, and the frequency and duration of app usage (see Table 6 and supplementary table 1, 2, 3, 4, 5, 6, 7, 8, 9, 10, 11 and 12). Gender did not affect app usage. Respondents in the clinical phase of their medical education were more likely to use medical education applications than those in the preclinical stage (43.2% vs. 27.4%). Respondents with a higher family household income, i.e., > RM10960, were more likely to use medical education and COVID-19 mHealth apps than those with lower household incomes.

**Table 6:** Demographic differences in overall mHealth app use

**Variable**		**Responses**	**Gender**				***p-*** **value**
			**Male**		**Female**	**Total**	
			**n (%)**		**n (%)**	**n (%)**	
**mHealth apps installed and used to find medical information**		Yes	25 (32.1)		64 (37.4)	89 (35.7)	.412
		No	53 (67.9)		107 (62.6)	160(64.3)	
**mHealth apps installed and used for health and fitness purposes**		Yes	30 (38.5)		67 (39.2)	97 (39.0)	.914
		No	48 (61.5)		104 (60.8)	152(61.0)	
**mHealth (apps) installed and used for COVID-19 management**		Yes	59 (75.6)		124 (72.5)	183(73.5)	.604
		No	19 (24.4)		47 (27.5)	66 (26.5)	
			**Phase of study**				***p-*** **value**
			**Preclinical (year 1–2)**		**Clinical (years 3–5)**	**Total**	
			***n*** ** = 117 (%)**		***n*** ** = 132 (%)**	***n*** ** = 249 (%)**	
**mHealth apps installed and used to find medical information**		Yes	32 (27.4)		57 (43.2)	89 (35.7)	.009
		No	85 (72.6)		75 (56.8)	160 (64.3)	
**mHealth apps installed and used for health and fitness purposes**		Yes	39 (33.3)		58 (43.9)	97 (39.0)	.087
		No	78 (66.7)		74 (56.1)	152 (61.0)	
**mHealth (apps) installed and used for COVID-19 management**		Yes	86 (73.5)		97 (73.5)	183 (73.5)	.997
		No	31 (26.5)		35 (26.5)	66 (26.5)	
			**Household income**				***p*** **-value**
			** < RM4849 per month (B40)**	**RM4850–10,959 per month (M40)**	** > RM10,960 per month (T20)**	**Total**	
			***n*** ** = 78 (%)**	***n*** ** = 121(%)**	***n*** ** = 49 (%)**	***n*** ** = 249 (%)**	
**mHealth apps installed and used to find medical information**		Yes	19 (24.7)	49 (39.8)	21 (42.9)	89 (35.7)	.048
		No	58 (75.3)	74 (60.2)	28 (57.1)	160 (64.3)	
**mHealth apps installed and used for health and fitness purposes**		Yes	23 (29.9)	54 (43.9)	20 (40.8)	97 (39.0)	.135
		No	54 (70.1)	69 (56.1)	29 (59.2)	152 (61.0)	
**mHealth (apps) installed and used for COVID-19 management**		Yes	49 (63.6)	94 (76.4)	40 (81.6)	183 (73.5)	.049
		No	28 (36.4)	29 (23.6)	9 (18.4)	66 (26.5)	

Female respondents (20.3%) used medical education apps more frequently (at least once per day) than males (8%). Usage frequency did not differ between those in the clinical and nonclinical phases of the study. However, those in the clinical phase showed a longer duration of medical education app use (more than two years). Although respondents with higher household incomes were more likely to use COVID-19 apps, they used them less frequently (17.5% at least once a day) than those with poorer financial support (81%).

### Demographic differences in-app awareness and attitudes

Older respondents (54.5%) and those in the clinical phase of their medical education (60.4%) were more aware of medical education applications. However, age and phase of study did not affect awareness of health and fitness or COVID-19 applications. None of the other demographic features measured were correlated with the respondents’ attitudes toward the three categories of applications (Supplementary Table 13, 14, 15, 16, 17, 18, 19, 20 and 21).

## Discussion

The use of mobile health technology is important in the field of medical education [39]. The COVID-19 pandemic has contributed to the growth of mHealth applications, increased their necessity, and altered usage patterns among the medical students [40]. This is the first cross-sectional study investigating the awareness of, attitudes toward, and usage of mobile health applications among medical students, including medical education, health and fitness, and COVID-19 management apps. We also examined obstacles to the use of mHealth apps and investigated the association among respondents’ demographic profiles, awareness and attitude toward their use of the mentioned apps.

The demographics of our respondents were comparable with those of previous studies in terms of student diversity [41]. Medical students of different races, with different financial backgrounds, and phases of medical education were included. More than two-thirds of our respondents were women, which is consistent with the overall percentage of female medical students enrolled in our university. Women were overrepresented, as has been the case in similar medical school surveys [32, 36, 42]. Hence, our findings are relevant to those of learning centers with similar environments and multiethnic populations.

In line with the result of previous studies, we found that medical students have good awareness of and attitudes toward mHealth apps [28, 41]. This might be due to the high number of individuals own mobile devices because of urbanization and the improved socioeconomic environment in Malaysia. The high number of smartphone ownership was consistent with the global trend, especially during the COVID-19 pandemic. Public health awareness is a vital contributor to the adoption of mHealth technologies [43]. As previously suggested, medical students are more likely to be aware of mHealth applications [28]. Hence, our students seemed ready to embrace this technology to promote effective learning, health and fitness and to improve their social connectivity [34–36, 44, 45].

The patterns of mHealth app usage among our respondents were noteworthy. The current pandemic is obviously the reason underlying the higher usage of COVID-19 management apps than fitness and medical education apps. Research has indicated the importance of a solid national regulatory framework to drive digital health [46]. The use of mHealth app is expected to increase during the pandemic because of the need for self-monitoring, health management, vaccination appointment booking, and contact tracing [12]. Although the pandemic could be a potential catalyst for mHealth adoption, another study including a French population observed that respondents did not trust the data protection and efficacy of these apps during the pandemic, highlighting the different cultural views on this matter [42].

Compared with global usage of mHealth app, our study demonstrated infrequent use of medical education and health and fitness apps [6, 32], despite the high mobile device ownership. This may indicate a preference for textbooks over medical apps as learning tools. A previous study revealed that medical students preferred face-to-face lectures and books to medical apps [47]. Most of our respondents indicated that uncertainty regarding which mHealth apps to choose was an obstacle to their use. Based on this, it can be inference that the lack of guidance from the medical curriculum could be an important contributor to low usage of medical education mHealth apps [48]. Our findings indicate that financial status affects the use of mHealth apps. Better financial status increased the likelihood of medical education app use. It indicates that the subscription costs of mobile applications are likely to be an obstacle to more widespread use. Increased app use was observed among students in the clinical phase of their medical education (year three onward in their medical program). They were more aware of mHealth apps, which is consistent with the results of other studies [6]. This suggests that earlier exposure to the clinical field may aid the integration of mHealth into medical students’ learning processes.

Our study demonstrated awareness about health and fitness mHealth apps and favorable attitudes toward them but low adoption of fitness apps among respondents. A systematic review of studies conducted among the general public revealed a similar phenomenon, illustrating the importance of identifying the obstacles to app usage [49]. High attrition rates among fitness apps users might be explained by the lack of entertainment or reward provided by exercise [50]. A higher percentage of those who do not use fitness apps among the lower-income group (70.1%) confirm that cost is an important barrier [51]. Although students can afford mobile devices, the cost of apps was regarded by many as an obstacle to the use of medical education and fitness apps in our study. A systematic review found the cost to be a leading factor in low mHealth usage in developing countries [52]. Hence, subscription or in-app purchase requirements could significantly hinder the use of mHealth app.

In contrast with previous research findings, there was no gender inequity in mHealth awareness, attitude, or use among our respondents [42, 53]. However, this was a general assessment of app use and not focused on a specific app. Additionally, respondents of both genders were enrolled under the same medical program. These factors may explain the homogeneity of our results in term of genders. Although there was no inequity in terms of usage, women were more likely to use the technology frequently once they adopted it. This could be due to differences in preferred revision and learning methods.

Age and phase of the study were significantly associated with the respondents’ awareness about and attitudes toward medical education apps. This may be due to the greater need for quick access to accurate disease management information in practice or when assessing patients [54]. Despite positive attitudes toward mHealth apps, our respondents found low usage rates of optional mHealth apps were found in all our respondents. Previous studies indicate that this may be due to the busy schedules of medical students [28, 54]. However, those in the clinical phase of their medical training spent most of their learning time in hospitals rather than in lecture halls or study rooms. Therefore, using mHealth medical education apps to access medical information has practical value for these students [33]. In addition to not knowing which app to use and the associated expense, concerns regarding user privacy were another significant barrier to using mHealth apps. Although today’s smartphones have various security features such as password lock, antimalware apps, and data encryption [55], research suggests low utilization of these security features [56]. Other security concerns include low confidence in in-app security and data protection [14].

The introduction of digital health courses, including mHealth apps, in a medical education program has recently increased worldwide, especially after the COVID-19 pandemic [29]. This study provides information on the awareness of, attitude toward, and practices of medical students as well as the obstacles to the use of mHealth apps. The result of this study will be useful for university administrators and the other relevant authorities as a basis for taking appropriate actions to eliminate the reported obstacles and facilitate the increased adoption of mHealth apps among students in a timely manner.

## Conclusion

Our study demonstrated the high awareness of mHealth apps among medical students, including different categories of apps (medical education, health and fitness, and COVID-19 management apps). Although medical students had a positive attitude toward mHealth implementation, the practice of mHealth usage was relatively low. Thus, addressing barriers to it use, such as high cost, security concerns, and uncertainty regarding which apps to choose, is essential to promote the use. Promoting the use of mHealth app by medical students for educative purposes could facilitate their future implementation in healthcare settings.

### Study limitations

There were several limitations to this study. First, our respondents were predominantly women, which may limit our findings’ applicability to both genders. Additionally, the study was a cross-sectional survey involving undergraduate medical students from UNIMAS, so the results may not fully represent Malaysian medical students. There is also potential recall bias and non-response in this type of study design, which may result in bias in the outcome measures [57]. Finally, the study was conducted during the COVID-19 pandemic, when most medical students received instructions via online distance learning. This interfered with our survey period as the distribution and return of surveys took longer than it might be in face-to-face. It may have led to misinterpretation of survey questions as no face-to-face explanations were possible.

## Supplementary Information

**Additional file 1**: **Table S1**. Frequency of medical apps usage by gender. **Table S2**. Frequency of health and fitness apps usage by gender. **Table S3. **Frequency of COVID-19 management apps usage by gender. **Table S4. **Frequency of medical education apps usage by Household Income. **Table S5. **Frequency of Health and Fitness apps usage by Household Income. **Table S6. **Frequency of COVID-19 Management Health apps usage by Household Income. **Table S7. **Frequency of medical apps usage by Phase of Study. **Table S8. **Frequency of health and fitness apps usage by Phase of Study. **Table S9.** Frequency of COVID-19 management apps usage by Phase of Study. **Table S10. **Practice of mHealth by Gender. **Table S11. **Practice of mHealth by Phase of Study. **Table S12. **Practice of mHealth by Household Income. **Table S13. **Mean age and mean attitude score by types of app. **Table S14. **Mean attitude score by gender.** Table S15. **Mean attitude score by phase of study. **Table S16. **Mean attitude score by household income. **Table S17. **Mean attitude score by ethnicity. **Table S18. **Mean age and awareness by types of app. **Table S19. **Awareness towards apps by gender. **Table S20. **Awareness towards apps by phase of study. **Table S21. **Awareness towards apps by ethnicity.

## Data Availability

The data and materials from this study are available from the corresponding author upon reasonable request.

## Abbreviations

4IR: Fourth Industrial Revolution
3G and 4G: Third- and fourth-generation mobile telecommunication
Apps: Applications
B40: Household income < RM4849 per month
CA: Cronbach’s alpha
COVID-19: Coronavirus disease 2019
eHealth: Electronic health
GB: Gigabyte
GPS: Global Positioning System
GPRS: General Packet Radio Service
IMS: Intercontinental Medical Statistics
IQR: Median
IT: Information technology
KKM: Kementerian Kesihatan Malaysia
M40: Household income of RM4850–10,959 per month
MCO: Movement Control Order
mHealth: Mobile health
MREC: Medical Research and Ethics Committee
n: Frequency
NMRR: National Medical Research Register
PLI: Poverty line income
SD: Standard deviation
SOPs: Standard of procedures
SPSS: Statistical Package for the Social Sciences software
T20: Household income > RM10960 per month
UNIMAS: Universiti Malaysia Sarawak
