# Supplementary material for: Mobile health applications: awareness, attitudes, and practices among medical students in Malaysia
Source: BMC Med Educ. 2022 Jul 15;22:544. doi: 10.1186/s12909-022-03603-4 (PMC9282901; doi:10.1186/s12909-022-03603-4)
Supplement: Supplementary file 1 — Additional file 1: Table S1. Frequency of medical apps usage by gender. Table S2. Frequency of health and fitness apps usage by gender. Table S3. Frequency of COVID-19 management apps usage by gender. Table S4. Frequency of medical education apps usage by Household Income. Table S5. Frequency of Health and Fitness apps usage by Household Income. Table S6. Frequency of COVID-19 Management Health apps usage by Household Income. Table S7. Frequency of medical apps usage by Phase of Study. Table S8. Frequency of health and fitness apps usage by Phase of Study. Table S9. Frequency of COVID-19 management apps usage by Phase of Study. Table S10. Practice of mHealth by Gender. Table S11. Practice of mHealth by Phase of Study. Table S12. Practice of mHealth by Household Income. Table S13. Mean age and mean attitude score by types of app. Table S14. Mean attitude score by gender. Table S15. Mean attitude score by phase of study. Table S16. Mean attitude score by household income. Table S17. Mean attitude score by ethnicity. Table S18. Mean age and awareness by types of app. Table S19. Awareness towards apps by gender. Table S20. Awareness towards apps by phase of study. Table S21. Awareness towards apps by ethnicity. [file 12909_2022_3603_MOESM1_ESM.docx]

**Supplementary Data**

**Table S1. Frequency of medical apps usage by gender**

**Supplementary Table 1: Frequency of medical apps usage by gender**

| Variable | Response | Gender | | | *p-value* |
| --- | --- | --- | --- | --- | --- |
|  |  | **Male**  **n= 25 (%)** | **Female**  **n= 64 (%)** | **Total**  **n= 89 (%)** |  |
| Frequency of medical education app(s) usage  Duration of medical education app(s) usage | At least once a day  At least once a week  At least once a month  Less than once per month  Less than 6 months  6 months to 2 years  More than 2 years | 2 (8.0)  22 (88.0)  1 (4.0)  0 (0.0)  4 (16.0)  11 (44.0)  10 (40.0) | 13 (20.3)  35 (54.7)  11 (17.2)  5 (7.8)  18 (28.1)  33 (51.6)  13 (20.3) | 15 (16.9)  57 (64.0)  12 (13.5)  5 (5.6)  22 (24.7)  44 (49.4)  23 (25.8) | .029  .137 |

**Table S2. Frequency of health and fitness apps usage by gender**

**Supplementary Table 2: Frequency of health and fitness apps usage by gender**

| Variable | Response | Gender | | | *p-value* |
| --- | --- | --- | --- | --- | --- |
|  |  | **Male**  **n= 30 (%)** | **Female**  **n= 67 (%)** | **Total**  **n= 97 (%)** |  |
| Frequency of health and fitness app(s)usage  Duration of health and fitness app(s)usage | At least once a day  At least once a week  At least once a month  Less than once per month  Less than 6 months  6 months to 2 years  More than 2 years | 10 (33.3)  17 (56.7)  1 (3.3)  2 (6.7)  7 (23.3)  21 (70.0)  2 (6.7) | 26 (38.8)  25 (37.3)  7 (10.4)  9 (13.4)  22 (32.8)  32 (47.8)  13 (19.4) | 36(37.1)  42 (43.3)  8 (8.2)  11 (11.3)  29 (29.9)  53 (54.6)  15 (15.5) | .254  .097 |

**Table S3. Frequency of COVID-19 management apps usage by gender**

**Supplementary Table 3: Frequency of COVID-19 management apps usage by gender**

| Variable | Response | Gender | | | *p-value* |
| --- | --- | --- | --- | --- | --- |
|  |  | **Male**  **n= 59 (%)** | **Female**  **n= 124 (%)** | **Total**  **n= 183 (%)** |  |
| Frequency of COVID-19 management health app(s) usage | At least once a day  At least once a week  At least once a month  Less than once per month | 15 (25.4)  31 (52.5)  13 (22.0)  0 (0.0) | 41 (33.1)  55 (44.4)  24 (19.4)  4 (3.2) | 56 (30.6)  86 (47.0)  37 (20.2)  4 (2.2) | .337 |

**Table S4. Frequency of medical education apps usage by Household Income**

**Supplementary Table 4: Frequency of medical education apps usage by Household Income**

| Variable | Response | Household Income | | | | *p-value* |
| --- | --- | --- | --- | --- | --- | --- |
|  |  | <RM4849 per month (B40)  n= 19 (%) | RM 4850-10959 per month (M40)  n= 49 (%) | >Rm10960 per month (T20)  n= 21 (%) | Total  n= 89 (%) |  |
| Frequency of medical education app(s) usage  Duration of medical education app(s) usage | At least once a day  At least once a week  At least once a month  Less than once per month  Less than 6 months  6 months to 2 years  More than 2 years | 3 (15.8)  10 (52.6)  3 (15.8)  3 (15.8)  8 (42.1)  6 (31.6)  5 (26.3) | 7 (14.3)  31 (65.3)  8 (16.3)  2 (4.1)  12 (24.5)  27 (55.1)  10 (20.4) | 5 (23.8)  15 (71.4)  1 (4.8)  0 (0.0)  2 (9.5)  11 (52.4)  8 (38.1) | 15 (16.9)  57 (64.0)  12 (13.5)  5 (5.6)  22 (24.7)  44 (49.4)  23 (25.8) | .314  .104 |

**Table S5. Frequency of Health and Fitness apps usage by Household Income**

**Supplementary Table 5: Frequency of Health and Fitness apps usage by Household Income**

| Variable | Response | Household Income | | | | *p-value* |
| --- | --- | --- | --- | --- | --- | --- |
|  |  | <RM4849 per month (B40)  n=23 (%) | RM 4850-10959 per month (M40)  n= 54 (%) | >Rm10960 per month (T20)  n= 20 (%) | Total  n= 97 (%) |  |
| Frequency of health and fitness app(s)usage  Duration of health and fitness app(s)usage | At least once a day  At least once a week  At least once a month  Less than once per month  Less than 6 months  6 months to 2 years  More than 2 years | 8 (34.8)  9 (39.1)  3 (13.0)  3 (13.0)  9 (39.1)  11 (47.8)  3 (13.0) | 20 (37.0)  24 (44.4)  3 (5.6)  7 (13.0)  14 (25.9)  31 (57.4)  9 (16.7) | 8 (40.0)  9 (45.0)  2 (10.0)  1 (5.0)  6 (30.0)  11 (55.0)  3 (15.0) | 36 (37.1)  42 (43.3)  8 (8.2)  11 (11.3)  29 (29.9)  53 (54.6)  15 (15.5) | .883  .869 |

**Table S6. Frequency of COVID-19 Management Health apps usage by Household Income**

**Supplementary Table 6: Frequency of COVID-19 Management Health apps usage by Household Income**

| Variable | Response | Household Income | | | | *p-value* |
| --- | --- | --- | --- | --- | --- | --- |
|  |  | <RM4849 per month (B40)  n=49 (%) | RM 4850-10959 per month (M40)  n=94 (%) | >Rm10960 per month (T20)  n= 40 (%) | Total  n= 183(%) |  |
| Frequency of COVID-19 management health app(s) usage | At least once a day  At least once a week  At least once a month  Less than once per month | 25 (51.0)  19 (38.8)  2 (4.1)  3 (6.1) | 24 (25.5)  47 (50.0)  23 (24.5)  0 (0.0) | 7 (17.5)  20 (50.0)  12 (30.0)  1 (2.5) | 56 (30.6)  86 (47.0)  37 (20.2)  4 (2.2) | .000 |

**Table S7. Frequency of medical apps usage by Phase of Study**

**Supplementary Table 7: Frequency of medical apps usage by Phase of Study**

| Variable | Response | Phase of Study | | | *p-value* |
| --- | --- | --- | --- | --- | --- |
|  |  | Preclinical Years  (Year 1 - 2)  n= 32 (%) | Clinical Years  (Year 3-5)  n= 57 (%) | Total  n= 89 (%) |  |
| Frequency of medical education app(s) usage  Duration of medical education app(s) usage | At least once a day  At least once a week  At least once a month  Less than once per month  Less than 6 months  6 months to 2 years  More than 2 years | 3 (9.4)    23 (71.9)  5 (15.6)  1 (3.1)  15 (46.9)  16 (50.0)    1 (3.1) | 12 (21.1)  34 (59.6)  7 (12.3)  4 (7.0)  7 (12.3)  28 (49.1)  22 (38.6) | 15 (16.9)  57 (64.0)  12 (13.5)  5 (5.6)  22 (24.7)    44 (49.4)  23 (25.8) | .476  <.001 |

**Table S8. Frequency of health and fitness apps usage by Phase of Study Supplementary Table 8: Frequency of health and fitness apps usage by Phase of Study**

| Variable | Response | Phase of Study | | | *p-value* |
| --- | --- | --- | --- | --- | --- |
|  |  | Preclinical Years  (Year 1 - 2)  n= 39 (%) | Clinical Years  (Year 3-5)  n= 58 (%) | Total  n= 97 (%) |  |
| Frequency of health and fitness app(s)usage  Duration of health and fitness app(s)usage | At least once a day  At least once a week  At least once a month  Less than once per month  Less than 6 months  6 months to 2 years  More than 2 years | 13 (33.3)  19 (48.7)  5 (12.8)  2 (5.1)  17 (43.6)  16 (41.0)  6 (15.4) | 23 (39.7)  23 (39.7)  3 (5.2)  9 (15.5)  12 (20.7)    37 (63.8)  9 (15.5) | 36 (37.1)  42 (43.3)    8 (8.2)  11 (11.3)  29 (29.9)  53 (54.6)  15 (15.5) | .214  .043 |

**Table S9. Frequency of COVID-19 management apps usage by Phase of Study**

**Supplementary Table 9: Frequency of COVID-19 management apps usage by Phase of Study**

| Variable | Response | Phase of Study | | | *p-value* |
| --- | --- | --- | --- | --- | --- |
|  |  | Preclinical Years  (Year 1 - 2)  n= 86 (%) | Clinical Years  (Year 3-5)  n= 97 (%) | Total  n=183 (%) |  |
| Frequency of COVID-19 management health app(s) usage | At least once a day  At least once a week  At least once a month  Less than once per month | 18 (20.9)  46 (53.5)  19 (22.1)  3 (3.5) | 38 (39.2)    40 (41.2)    18 (18.6)  1 (1.0) | 56 (30.6)  86 (47.0)  37 (20.2)  4 (2.2) | .039 |

**Table S10. Practice of mHealth by Gender**

**Supplementary Table 10: Practice of mHealth by Gender**

| Variable | Responses | Gender | | | *p-value* |
| --- | --- | --- | --- | --- | --- |
|  |  | **Male**  **n=78 (%)** | **Female**  **n=171(%)** | **Total**  **n= 249(%)** |  |
| Medical education app(s) used  Health and fitness app(s) used  COVID-19 management health apps used | UpToDate  Medscape  DynaMed  Oxford Clinical Handbook  Epocrates  Lexicomp  Bluebook/ Drug Doses (Frank Shann) on 8  QxMD calculator / MDCalc calculator  Others  Physical activity  food trackers  Fasting Applications  Others  MySejahtera  COVIDtrace  Qmunity  My Trace | 3 (12.0)  22 (88.0)  3 (12.0)  7 (28.0)  6 (24.0)  6 (24.0)  0 (0.0)  2 (8.0)  8 (32.0)  30 (100.0)  10 (33.3)  3 (10.0)  0 (0.0)  59 (100.0)  4 (6.8)  10 (16.9)  1 (1.7) | 18 (28.1)  60 (93.8)  5 (7.8)  24 (37.5)  10 (15.6)  3 (4.7)  6 (9.4)  4 (6.3)  20 (31.3)  57 (85.1)  28 (41.8)  15 (22.4)  2 (3)  123 (99.2)  12 (9.7)  23 (18.5)  3 (2.4) | 21 (23.6)  82 (92.1)  8 (9.0)  31 (34.8)  16 (18.0)  9 (10.1)  6 (6.7)  6 (6.7)  28 (31.5)  87 (89.7)  38 (39.2)  18 (18.6)  2 (2.1)  182 (99.5)  16 (8.7)  33 (18.0)  4 (2.2) | .029  .254  .337 |

**Table S11. Practice of mHealth by Phase of Study**

**Supplementary Table 11: Practice of mHealth by Phase of Study**

| Variable | Responses | Phase of Study | | | *p-value* |
| --- | --- | --- | --- | --- | --- |
|  |  | Preclinical Years  (Year 1 - 2)  n= 117 (%) | Clinical Years  (Year 3-5)  n= 132 | Total  249 (%) |  |
| Medical education app(s) used  Health and fitness app(s) used  COVID-19 management health apps used | Uptodate  Medscape  DynaMed  Oxford Clinical Handbook  Epocrates  Lexicomp  Bluebook/ Drug Doses (Frank Shann) on 8  QxMD calculator / MDCalc calculator  Others    Physical activity  food trackers  Fasting Applications  Others  MySejahtera  COVIDtrace  Qmunity  My Trace | 3 (9.4)  30 (93.8)  0 (0.0)  4 (12.5)  1 (3.1)  1 (3.1)  0 (0.0)  0 (0.0)  9 (28.1)  36 (92.3)  9 (23.1)  8 (20.5)  0 (0.0)  85 (98.8)  8 (9.3)  12 (14.0)  1 (1.2) | 18 (31.6)  52 (91.2)  8 (14.0)  27 (47.4)  15 (26.3)    8 (14.0)  6 (10.5)  6 (10.5)  19 (33.3)  51 (87.9)  29 (50.0)  10 (17.2)  2 (3.4)  97 (100)  8 (8.2)  21 (21.6)  3 (3.1) | 21 (23.6)  82 (92.1)  8 (9.0)  31 (34.8)  16 (18.0)  9 (10.1)  6 (6.7)  6 (6.7)  28 (31.5)  87 (89.7)  38 (39.2)  18 (18.6)  2 (2.1)  182 (99.5)  16 (8.7)  33 (18.0)  4 (2.2) | .476  .214  .039 |

**Table S12. Practice of mHealth by Household Income**

**Supplementary Table 12: Practice of mHealth by Household Income**

| Variable | Responses | Household Income | | | | *p-value* |
| --- | --- | --- | --- | --- | --- | --- |
|  |  | <RM4849 per month (B40)  n=78 (%) | RM 4850-10959 per month (M40)  n=121(%) | >Rm10960 per month (T20)  n= 49 (%) | Total  n= 249 (%) |  |
| Medical education app(s) used  Health and fitness app(s) used  COVID-19 management health apps used | Uptodate  Medscape  DynaMed  Oxford Clinical Handbook  Epocrates  Lexicomp  Bluebook/ Drug Doses (Frank Shann) on 8  QxMD calculator /MDCalc calculator  Others  Physical activity  food trackers  Fasting Applications  Others  MySejahtera  COVIDtrace  Qmunity  My Trace | 9 (47.4)  18 (94.7)  0 (0.0)  7 (36.8)  0 (0.0)  0 (0.0)  1 (5.3)  2 (10.5)  7 (36.8)  21 (91.3)  7 (30.4)  3 (13)  0 (0.0)  49 (100.0)  5 (10.2)  9 (18.4)  2 (4.1) | 8 (16.3)  44 (89.8)  4 (8.2)  11 (22.4)  10 (20.4)  5 (10.2)  2 (4.1)  2 (4.1)  17 (34.7)  47 (87.0)  24 (44.4)  12 (22.2)  2 (3.7)  93 (98.9)  9 (9.6)  17 (18.1)  1 (1.1) | 4 (19.0)  20 (95.2)  4 (19.0)  13 (61.9)  6 (28.6)  4 (19.0)  3 (14.3)  2 (9.5)  4 (19.0)  19 (95.0)  7 (35.0)  3 (15.0)  0 (0.0)  40 (100.0)  2 (5.0)  7 (17.5)  1 (2.5) | 21 (23.6)  82 (92.1)  8 (9.0)  31 (34.8)  16 (18.0)  9 (10.1)  6 (6.7)  6 (6.7)  28 (31.5)  87 (89.7)  38 (39.2)  18 (18.6)  2 (2.1)  182 (99.5)  16 (8.7)  33 (18.0)  4 (2.2) | .314  .883  .000 |

**Table S13. Mean age and mean attitude score by types of app**

**Supplementary Table 13: Mean age and mean attitude score by types of app**

| Variable | Mean Age | Mean Attitude Score | *p-value* |
| --- | --- | --- | --- |
| Medical education app(s) used  Health and fitness app(s) used  COVID-19 management health apps used | 21.6 ± 1.5 | 3.86 ± 0.68  3.93 ± 0.63  4.14 ± 0.64 | .886  .916  .462 |

**Table S14. Mean attitude score by gender**

**Supplementary Table 14: Mean attitude score by gender**

| Variable | Gender | | *p-value* |
| --- | --- | --- | --- |
|  | **Male** | **Female** |  |
| Medical education app(s) used  Health and fitness app(s) used  COVID-19 management health apps used | 3.81  3.93  4.17 | 3.88  3.93  4.13 | .462  .979  .688 |

**Table S15. Mean attitude score by phase of study**

**Supplementary Table 15: Mean attitude score by phase of study**

| Variable | Phase of Study | | *p-value* |
| --- | --- | --- | --- |
|  | **Preclinical Years**  **(Year 1-2)** | **Clinical Years**  **(Year 3-5)** |  |
| Medical education app(s) used  Health and fitness app(s) used  COVID-19 management health apps used | 3.83 ± 0.74  3.95 ± 0.65  4.20 ± 0.63 | 3.89 ± 0.62  3.92 ± 0.62  4.09 ± 0.64 | .564  .721  .155 |

**Table S16. Mean attitude score by household income**

**Supplementary Table 16: Mean attitude score by household income**

| Variable | Household Income | | | *p-value* |
| --- | --- | --- | --- | --- |
|  | <RM4849 per month (B40) | RM 4850-10959 per month (M40) | >Rm10960 per month (T20) |  |
| Medical education app(s) used  Health and fitness app(s) used  COVID-19 management health apps used | 3.63 ± 0.79  3.91 ± 0.62  4.09 ± 0.58 | 3.68 ± 0.71  4.04 ± 0.56  4.04 ± 0.92 | 4.04 ± 0.82  4.15 ± 0.54  4.26 ± 0.51 | .001  .000  .162 |

**Table S17. Mean attitude score by ethnicity**

**Supplementary Table 17: Mean attitude score by ethnicity**

| Variable | Ethnicity | | |  |  | *p-value* |
| --- | --- | --- | --- | --- | --- | --- |
|  | Malay | Chinese | Sarawak Bumiputera | Indian | Others |  |
| Medical education app(s) used  Health and fitness app(s) used  COVID-19 management health apps used | 3.90 ± 0.58  3.98 ± 0.57  4.09 ± 0.57 | 3.69 ± 0.78  3.83 ± 0.73  4.09 ± 0.83 | 3.97 ± 0.58  3.97 ± 0.60  4.22 ± 0.47 | 4.06 ± 0.78  4.00 ± 0.70  4.28 ± 0.63 | 3.53 ± 1.51  3.68 ± 0.41  4.06 ± 0.59 | .063  .490  .571 |

**Table S18. Mean age and awareness by types of app**

**Supplementary Table 18: Mean age and awareness by types of app**

| Variable | Mean Age | Awareness to app | | *p-value* |
| --- | --- | --- | --- | --- |
|  |  | **Yes** | **No** |  |
| Medical education app(s) used  Health and fitness app(s) used  COVID-19 management health apps used | 21.6 ± 1.5 | 21.2 ± 1.54  21.5 ± 1.64  21.6 ± 1.70 | 21.8 ± 1.46  21.6 ± 1.49  21.6 ± 1.49 | .001  .543  .963 |

**Table S19. Awareness towards apps by gender**

**Supplementary Table 19: Awareness towards apps by gender**

| Variable | Awareness status | Gender | | *p-value* |
| --- | --- | --- | --- | --- |
|  |  | **Male**  **n (%)** | **Female**  **n (%)** |  |
| Medical education app(s) used  Health and fitness app(s) used  COVID-19 management health apps used | Yes  No  Yes  No  Yes  No | 52 (66.7)  26 (33.3)  58 (74.4)  20 (25.6)  64 (82.1)  14 (17.9) | 102 (59.6)  69 (40.4)  132 (77.2)  39 (22.8)  142 (83)  29 (17) | .029  .626  .848 |

**Table S20. Awareness towards apps by phase of study**

**Supplementary Table 20: Awareness towards apps by phase of study**

| Variable | Awareness status | Phase of study | | *p-value* |
| --- | --- | --- | --- | --- |
|  |  | **Preclinical years (Year 1-2)**  **n (%)** | **Clinical years**  **(Year 3-5)**  **n (%)** |  |
| Medical education app(s) used  Health and fitness app(s) used  COVID-19 management health apps used | Yes  No  Yes  No  Yes  No | 61 (52.1)  56 (47.9)  88 (75.2)  29 (24.8)  96 (82.1)  21 (17.9) | 93 (70.5)  39 (29.5)  102 (77.3)  30 (22.7)  110 (83.3)  22 (16.7) | .003  .703  .789 |

**Table S20. Awareness towards apps by ethnicity**

**Supplementary Table 21: Awareness towards apps by ethnicity**

| Variable | Awareness Status | Ethnicity | | |  |  | *p-value* |
| --- | --- | --- | --- | --- | --- | --- | --- |
|  |  | Malay | Chinese | Sarawak Bumiputera | Indian | Others |  |
| Medical education app(s) used  Health and fitness app(s) used  COVID-19 management health apps used | Yes  No  Yes  No  Yes  No | 48 (53.9)  41 (46.1%)  63 (70.8)  26 (29.2)  71 (79.8)  18 (20.2) | 51 (59.4)  20 (28.2)  57 (80.3)  14 (19.7)  58 (81.7)  13 (18.3) | 38 (59.4)  26 (40.6)  52 (81.3)  12 (18.8)  57 (89.1)  7 (10.9) | 14 (77.8)  4 (22.2)  15 (83.3)  3 (16.7)  14 (77.8)  4 (22.2) | 3 (60)  2 (40)  2 (40)  3 (60)  4 (80)  1 (20) | .112  .128  .608 |
